# Hax1 regulate focal adhesion dynamics through IQGAP1

**DOI:** 10.1186/s12964-023-01189-y

**Published:** 2023-07-24

**Authors:** Xinyi Ren, Xiaopu Guo, Zihan Liang, Renxian Guo, Shaohui Liang, Han Liu

**Affiliations:** School of Basic Medical Sciences, Wenzhou Medical University, Wenzhou, 325035 Zhejiang China

**Keywords:** Cell migration, Focal Adhesion, IQGAP1, Hax1, Microtubules

## Abstract

Cell migration is a highly orchestrated process requiring the coordination between the cytoskeleton, cell membrane and extracellular matrix adhesions. Our previous study demonstrated that Hax1 interacts with EB2, a microtubule end-binding protein, and this interaction regulate cell migration in keratinocytes. However, little is known about the underlying regulatory mechanism. Here, we show that Hax1 links dynamic focal adhesions to regulate cell migration via interacting with IQGAP1, a multidomain scaffolding protein, which was identified by affinity purification coupled with LC–MS/MS. Biochemical characterizations revealed that C-terminal region of Hax1 and RGCT domain of IQGAP1 are the most critical binding determinants for its interaction. IQGAP1/Hax1 interaction is essential for cell migration in MCF7 cells. Knockdown of *HAX1* not only stabilizes focal adhesions, but also impairs the accumulation of IQGAP in focal adhesions. Further study indicates that this interaction is critical for maintaining efficient focal adhesion turnover. Perturbation of the IQGAP1/Hax1 interaction in vivo using a membrane-permeable TAT-RGCT peptide results in impaired focal adhesion turnover, thus leading to inhibition of directional cell migration. Together, our findings unravel a novel interaction between IQGAP1 and Hax1, suggesting that IQGAP1 association with Hax1 plays a significant role in focal adhesion turnover and directional cell migration.

Video Abstract

**Supplementary Information:**

The online version contains supplementary material available at 10.1186/s12964-023-01189-y.

## Introduction

Cell migration is a fundamental cellular process essential for developmental morphogenesis, wound healing, and tumor metastasis. This intricate process requires highly integrated orchestration between the cytoskeleton and the plasma membrane cortex [1, 2]. It is well documented that this process is dependent on focal adhesions (FAs) which is multi-protein complexes that link the extracellular matrix to the actin cytoskeleton [3–5]. Interestingly, previous findings demonstrated that peripheral FAs can be specifically targeted by microtubules (MTs). Furthermore, accumulating evidence has shown that growing microtubules can promote focal adhesion dynamics by serving as tracks to deliver proteins essential for focal adhesion turnover [6–8]. MT plus-end tracking proteins, referred to as + TIPs play a critical role during this process. Although many + TIPs have been identified, the most well-known + TIPs is the end-binding proteins (EB1, EB2, and EB3) [9]. However, unlike EB1 and EB3 which have been established to regulate MT dynamics by promoting MT growth and suppressing catastrophe [9, 10], EB2 does not play a direct role in MT dynamic instability [9]. Recently, more studies suggest that EB2 may function as an adaptor molecule to recruit signaling molecules to MTs, which is required for various cellular processes. For example, EB2 has been demonstrated to play an essential role in the regulation of focal adhesion dynamics and cell migration via its interaction with MAP4K4 [11]. Additionally, our recent work also indicated that EB2 plays a critical role in focal adhesion turnover and epidermal migration through its interaction with Hax1 [12].

HAX1 is a ubiquitous protein involved in the regulation of apoptosis, cell motility, and calcium homeostasis. Its overexpression was reported in several tumors [13–15], including breast cancer [16]. Previous reports have shown that Hax1 plays an essential role in the regulation of cell migration and adhesion. For example, Hax1 depletion in neutrophils enhances integrin-mediated cell adhesion and impaired directed migration [17]. In addition, our previous reports indicated that Hax1 depletion in skin epidermal cells stabilizes focal adhesions and impairs epidermal migration in vitro and in vivo and Hax1-EB2 complex plays an essential role in these processes [12]. However, the molecular mechanisms underlying these effects are still unclear and How EB2-Hax1 complex contributes to focal adhesion dynamics remains to be explored. To probe deeper into the role of Hax1 in cell migration, we performed tandem affinity purification to identify its binding partners, our LC–MS/MS analysis revealed an intriguing interaction partner, IQGAP1, which is critically involved in cytoskeleton remodeling and cell migration [18, 19].

IQ motif-containing GTPase-activating protein 1 (IQGAP1) is a multidomain scaffolding protein that essentially participated in several cellular events including cell–cell adhesion, cell migration and invasion. [20–23]. These domains mediate protein–protein interaction, which include a calponin homology domain (CHD), a region containing two tryptophans (WW), four IQ motifs (IQ), a Ras GTPase-activating protein-related domain (GRD) and a RasGAP C-terminus (RGCT) [18]. It’s well documented that IQGAP1, distributed in the leading edge of migrating cells, can rearrange actin filaments and regulate cell migration through directly interacting with the Rho family GTPases Cdc42 and Rac1 [24]. It can also interact with microtubule plus-end-tracking proteins (+ Tips) such as CLIP170 and SKAP to steer cell migration [25, 26]. In addition, IQGAP1 was identified in FAs [27, 28] and in focal complexes (FCs) of keratinocytes, where it binds to the integrin-linked kinase ILK [29]. However, whether IQGAP1 interacts with FA proteins or is directly involved in the regulation of FA dynamics is unknown.

In this report, we establish that the EB2-binding protein, Hax1, directly interacts with the cell cortex-distributed scaffold protein, IQGAP1, via its C terminus. The interaction between HAX1 and IQGAP1 plays an essential role in maintaining efficient focal adhesion turnover and regulating cell migration process of MCF7 and perturbation of the IQGAP1-HAX1 interaction impairs focal adhesion dynamics and inhibits cell migration. Furthermore, we find that Hax1 depletion dramatically reduces IQGAP1 in FAs and perturbation of the IQGAP1-HAX1 interaction also impairs IQGAP1 colocalization with peripheral FAs, suggesting Hax1 regulates localization and association of IQGAP1 to mature FAs and thereby controls FA dynamics. Taken together, our study unravels an important mechanism whereby MT plus end–directed transport of the Hax1–IQGAP1 complex regulates localization and association of IQGAP1 to mature FAs and thereby controls FA dynamics and cell migration.

### Experimental procedures

#### Antibodies and reagents

The following antibodies were obtained from commercial sources: anti-Hax1 mouse monoclonal antibody (BD Biosciences), anti-IQGAP1 rabbit polyclonal antibodies (Abcam), anti-myc rabbit polyclonal antibody (Thermo Fisher Scientific), mouse monoclonal antibody against vinculin was obtained from Sigma. Rabbit monoclonal antibody against HA and mouse monoclonal antibody against α-tubulin were obtained from Cell Signaling Technology. Microtubule-binding protein spin-down assay kit was obtained from Cytoskeleton (Denver, CO). Nocodazole was obtained from Sigma (St Louis, MO). Other chemicals or reagents were obtained from Thermo Fisher Scientific, unless otherwise indicated.

#### Plasmid DNA constructions

Hax1 N-terminal and C-terminal mutants were cloned into mammalian expression vectors pcDNA3.1 + N-HA as described previously [12]. The plasmid encoding full length IQGAP1 Cdna: pcDNA3-Myc-IQGAP1 was a gift from David Sacks (Addgene plasmid #30,118; http://n2t.net/addgene:30118; RRID:Addgene_30118) [30]. IQGAP1 N-terminal (residues 1–863), C-terminal (residues 864–1657), GRD domain (residues 1025–1238), RGCT domain (residues 1276–1657) and their mutants were cloned into mammalian expression vectors pcDNA3.1 + N-HA and pcDNA3.1 + N-Myc (with N-terminal Myc or HA tag). To generate TAT-GFP proteins to perturb the IQGAP1-Hax1 interaction, an 11-amino acid TAT sequence followed by a GFP gene were inserted into the pET-22b vector. Then RGCT amino acid sequence competing with the IQGAP1-Hax1 interaction was inserted between TAT and GFP sequence. All plasmid constructs were sequenced for verification.

#### Cell culture and transfections

HEK293T cells, from the American Type Culture Collection (ATCC, Manassas, VA), were cultured in DMEM medium supplemented with 10% fetal bovine serum (FBS) and 1% penicillin and streptomycin. MCF7 cells, from ATCC, were also maintained in DMEM medium containing 10% FBS with additional supplement of 10 μg/ml insulin. Knockdown of Hax1 and IQGAP1 in MCF7 cells using siRNA (5’-GGAUACGUUUCCACGAUAATT-3’) and siRNA (5’-UCCUAUGGUUGUGGUCCGAAATT-3’) synthesized by Tsingke Biotechnology (China) respectively. Cells were transfected with FuGENE HD (Promega), according to the manufacturer’s protocol. Cells were usually examined 24–48 h post-transfection.

#### Cell migration assays and time-lapse video microscopy

Scratch-wound healing assay was performed as described previously [12]. Briefly, cells were plated on 12 well plate until reached confluency. Wounds were created on the cell monolayer using a 200 μl pipette tip. The plates were then washed with PBS, replenished with media and photographed using a phase contrast microscope at indicated time points. For assays of single cell migration, cells were plated on fibronectin-coated glass-bottomed culture dishes and imaged with an Olympus IXplore SpinSR Super Resolution Confocal Microscope for 3 h at 1 frame per 10 min and tracked manually in ImageJ.

#### LC–MS/MS Analysis

For tandem affinity purification samples, protein samples were separated by SDS-PAGE. Specific protein bands were excised by sterile razor blade and chopped into small pieces. Each sample was washed in water, destained and digested with trypsin. The peptide mixtures were loaded to a Dionex Ultimate 3000 RSLCnano system (Thermo Scientific) and analyzed via electrospray tandem mass spectrometry (LC–MS/MS) on a Q-Exactive (Thermo scientific) mass spetrometer, using a 70,000 RP survey scan, m/z 350–1800.

#### Immunoprecipitation

Myc or HA-tagged protein-expressing 293 T cells were lysed in NP40 lysis buffer (1% NP40, 20 mM Tris–HCl pH8.0, 0.8% NaCl, 10% Glycerol) containing protease inhibitors on ice. Lysates were centrifuged by 13,000 rpm for 10 min at 4 °C and incubated with HA-agarose beads overnight at 4 °C. After five times washes, the beads were boiled in SDS-PAGE sample buffer for 5 min and separated using 10% SDS–polyacrylamide gel electrophoresis (PAGE) and electroblotted onto a NC membrane. The immunoblot was incubated with blocking buffer at room temperature for 1 h, followed by an overnight incubation with the primary antibody at 4 °C. Blots were washed three times with Tween-20/Tris-buffered saline (TBST) and incubated with a 1:1,000 dilution of secondary antibody for 1 h at room temperature. Blots were washed three times with TBST again and visualized with enhanced chemiluminescence (ECL) substrate.

#### Immunofluorescence

Cells were seeded on coverslips in 24-well plates for transfection or other treatment. Cells were fixed in 4% formaldehyde and then permeabilized in 0.1% Triton solution. After three times washes with PBS, cells were blocked with 1.5% bovine serum albumin at room temperature for 1 h, followed by an overnight incubation with the primary antibody at 4 °C. Blots were washed three times with PBST (0.02% Tween 20 in PBS) and incubated with a 1:250 dilution of secondary antibody for 1 h at room temperature. After five times washes with PBST, coverslips were mounted with Slow Fade mounting solution and sealed with nail polish.

#### Focal adhesion assembly/disassembly measurements

Cells were plated on fibronectin-coated dishes and transfected with plasmid encoding DsRed-Zyxin. Time series of images were acquired on a Nikon A1R N-SIM/N-STORM Super Resolution Microscope equipped with a × 100 SR Apo TIRF (1.49 oil) lens and an electron microscopy charge-coupled device camera. The rate constants for focal adhesion assembly and disassembly were obtained by calculating the slope of relative fluorescence intensity decreases of individual focal adhesion on a semilogarithmic scale against time as previously described [12].

#### Expression and purification of recombinant proteins

TAT-GFP-His fused proteins were expressed and purified as described previously [26]. Briefly, The fused protein was overproduced in *Escherichia coli* BL21(DE3) cells. Cells were grown in LB medium with 100 μg/ml ampicillin at 37℃ for 2.5 h until OD_600_ (optical density at 600 nm) reached value of 0.6. The culture was then induced with 0.2 mM isopropyl-b,D-thiogalactopyranoside at 28℃ for 4–6 h and harvested after induction. The collected bacteria were resuspended in ice-cold PBS and lysed with ultrasonication. Cell debris was removed by centrifugation and the harvested fused protein was purified with nickel-nitrilotriacetic acid-agarose beads (Thermo scientific).

To probe the effect of TAT-GFP fusion proteins on the IQGAP1-Hax1 interaction, MCF7 cells were cultured until 70–80% confluence and then starved for 7 h, followed by pretreatment of TAT-RGCT-GFP fusion proteins or TAT-GST-GFP as a parallel control at 2.5 μM for 1 h. After incubation, cells were washed with PBS and ready for further experiments.

#### Statistical analysis

Statistical analysis was performed using Excel, OriginPro, or GraphPad Prism software. Box plots are used to describe the entire population without assumptions on the statistical distribution. Statistical significance (*p* value) between two experimental conditions was assessed using Student’s *t* test. Differences were considered significant when *p* < 0.05.

## Results

### Identification of a novel Hax1-IQGAP1 complex in interphase cells

Our previous studies revealed the functional importance of Hax1 in focal adhesion dynamics and cell migration in keratinocytes [12]. Although the interaction between Hax1 and a MT plus-end tracking protein EB2 was identified of critical importance in focal adhesion turnover and cell migration in keratinocytes, microtubule plus-end dynamics remain unchanged in both Hax1 and EB2 depleted cells (Data not shown). These results suggest that Hax1-EB2 complex does not regulate MT dynamics or FA targeting, but instead more likely acts downstream of MTs to facilitate FA turnover.

First, to test specifically if the knockdown of Hax1 contributes to cell migration, we performed wound-healing assays. MCF7 cells were depleted of Hax1 by transfection with siRNA duplexes, followed by starvation and generated a linear scratch by a sterile pipette tip into the cell monolayer as previously described [12]. Western blotting analysis confirmed that Hax1 were efficiently depleted in MCF7 (Fig. S1B) cell line. Wound-healing scratch assays show that suppression of Hax1 protein significantly delayed the recovery of these scratches in MCF7 cell line (Fig. S1A). Our quantitative analyses indicate that suppression of Hax1 reduced relative migration by 50% compared with the control (Fig. S1C). To further assess Hax1 impact on cell motility at the single cell level, we used video microscopy to monitor the movement track and velocities of individual cells. As shown in Fig. S1D, suppression of Hax1 induced significant inhibition of MCF7 cell motility. Quantitative analyses show that compared with cells transfected with scrambled siRNA, the migration speed was significantly reduced in cells depleted with Hax1 (Fig. S1E). Together, our results confirm that Hax1 is essential for both collective and single cell migration in MCF7 cells, which is in consistent with our previous report on keratinocytes [12].

**Fig. 1:**
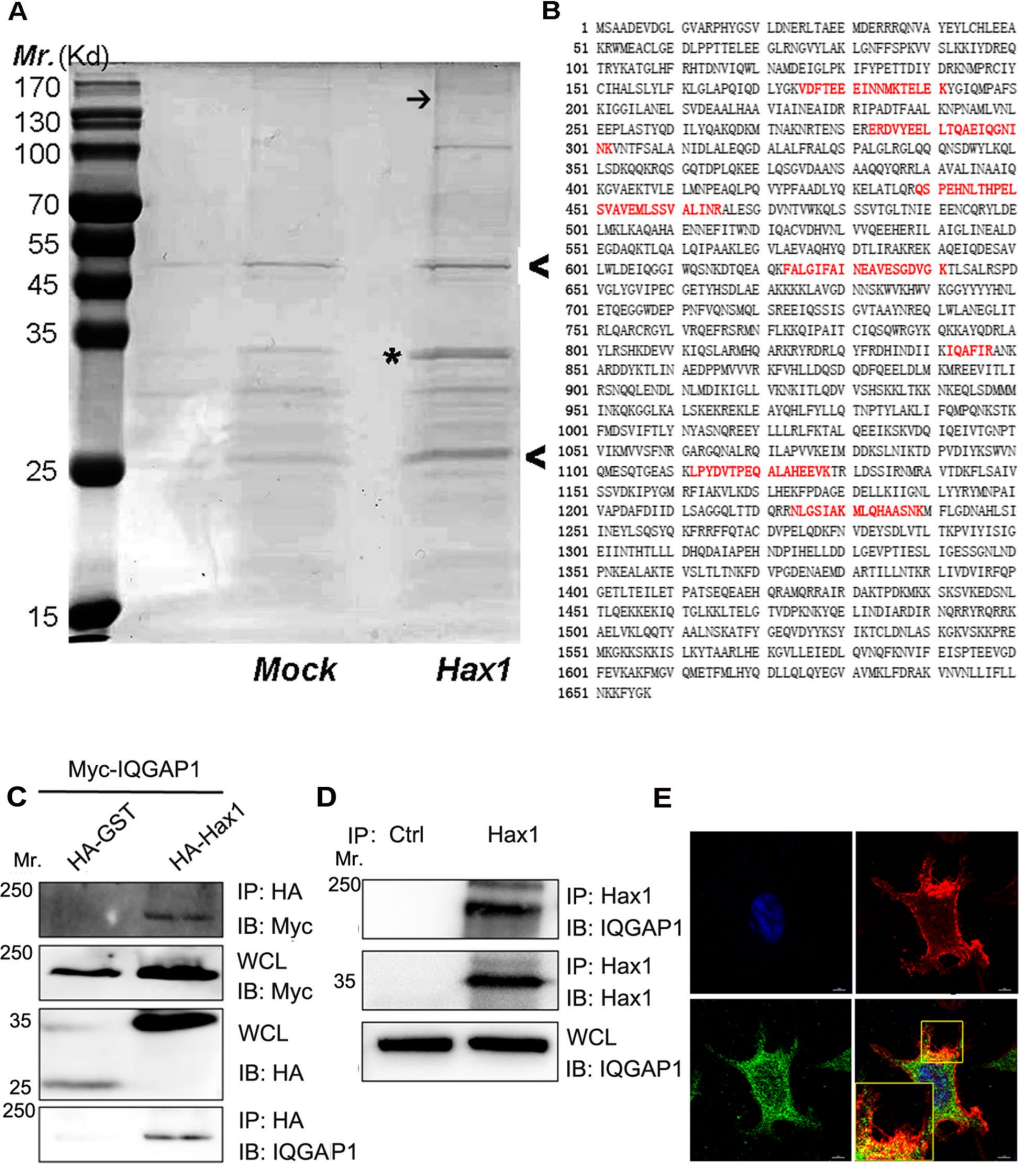
Identification of IQGAP1 as a novel Hax1 Binding Partners by Tandem Affinity Purification. **A**, Hax1 and its associated proteins were isolated by tandem affinity purification and resolved by SDS-PAGE. Immunoglobulin G heavy chain and light chain are marked by arrowheads. The putative band for Hax1 is marked by star. Putative band for IQGAP1 is marked by an arrow. **B**, IQGAP1 were found as Hax1 binding protein by mass spectrometric sequencing of proteins from the coomassie blue-stained gel. Identified matched peptides were shown in bold red. **C**, HEK293T cells were transfected with different plasmids as indicated. Cell lysates were immunoprecipitated (*IP*) with HA-agarose beads and immunoblotted (*IB*) with different antibodies as indicated. For whole-cell lysate (*WCL*), 10 μg of total protein was used. Note that only Hax1 specifically pulls down IQGAP1. **D**, Hax1 immunoprecipitates from MCF7 cells were immunoblotted with antibodies against IQGAP and Hax1. For whole-cell lysate (*WCL*), 10 μg of total protein was used. Note that Hax1 immunoprecipitation brought down IQGAP1. **E**, MCF7 cells were immunostained for IQGAP1 (red), Hax1 (green), and DAPI (blue). Scale bar, 10 μm. Cells were imaged by Nikon's Structured Illumination microscope. The boxed areas are magnified as insets. Note that Hax1 colocalizes with IQGAP1 signal in the protrusive region and accumulates at the leading edge of migrating cells

Now we’ve confirmed that Hax1 plays an essential role in migration of MCF7 cells. To further unravel the molecular mechanism and identify the Hax1-associated molecule involved in Hax1 regulated cell migration, we characterized Hax1-interacting proteins in anti-HA immunocomplexes isolated from HA-Hax1–transfected HEK293T cells using affinity purification coupled with LC–MS/MS. The purified Hax1 associated proteins were resolved in SDS-PAGE and examined by coomassie blue staining (Fig. 1A). We identified several potential binding partners of Hax1, including EB2, HSPs, myosin, AP-2, and IQGAP1. Among all these proteins, EB2 is the binding protein of Hax1 that we’ve confirmed in our previous study [12], HSPs and myosins are common contaminant proteins detectable by mass spectrometry, whereas IQGAP1 (Fig. 1B) is an interesting candidate that we selected for further study, because it was well documented that IQGAP1 is essentially involved in cytoskeleton remodeling and cell migration [21]. To verify the interaction between Hax1 and IQGAP1, we co-expressed Hax1 and IQGAP1 in 293 T cells. Immunoprecipitation results confirm that IQGAP1 was present in the Hax1 immunoprecipitates (Fig. 1C). In endogenous co-immmunoprecipitation assays, we also observed a significant amount of IQGAP1 in Hax1 immunoprecipitates but not in the control samples in MCF7 cells (Fig. 1D). IQGAP1 is a scaffold protein mainly distributed in the leading edge of migrating cells. The identification of IQGAP1/Hax1 protein complex prompted us to examine potential co-localization of Hax1 and IQGAP1 in the leading edge of cells. Immunofluoresence staining of Hax1 and IQGAP1 in MCF7 cells showed that although Hax1 signal distributed throughout the whole cell, it enriched at the cell cortex and superimposed onto that of the IQGAP1 signal (Fig. 1E). Thus, we conclude that Hax1 interacts and co-distributes with IQGAP1 at the cell cortex in leading edge of cells. Together, we conclude that IQGAP1 associates with Hax1 as protein complex.

### Characterization of molecular interactions between Hax1 and IQGAP1

**Fig. 2:**
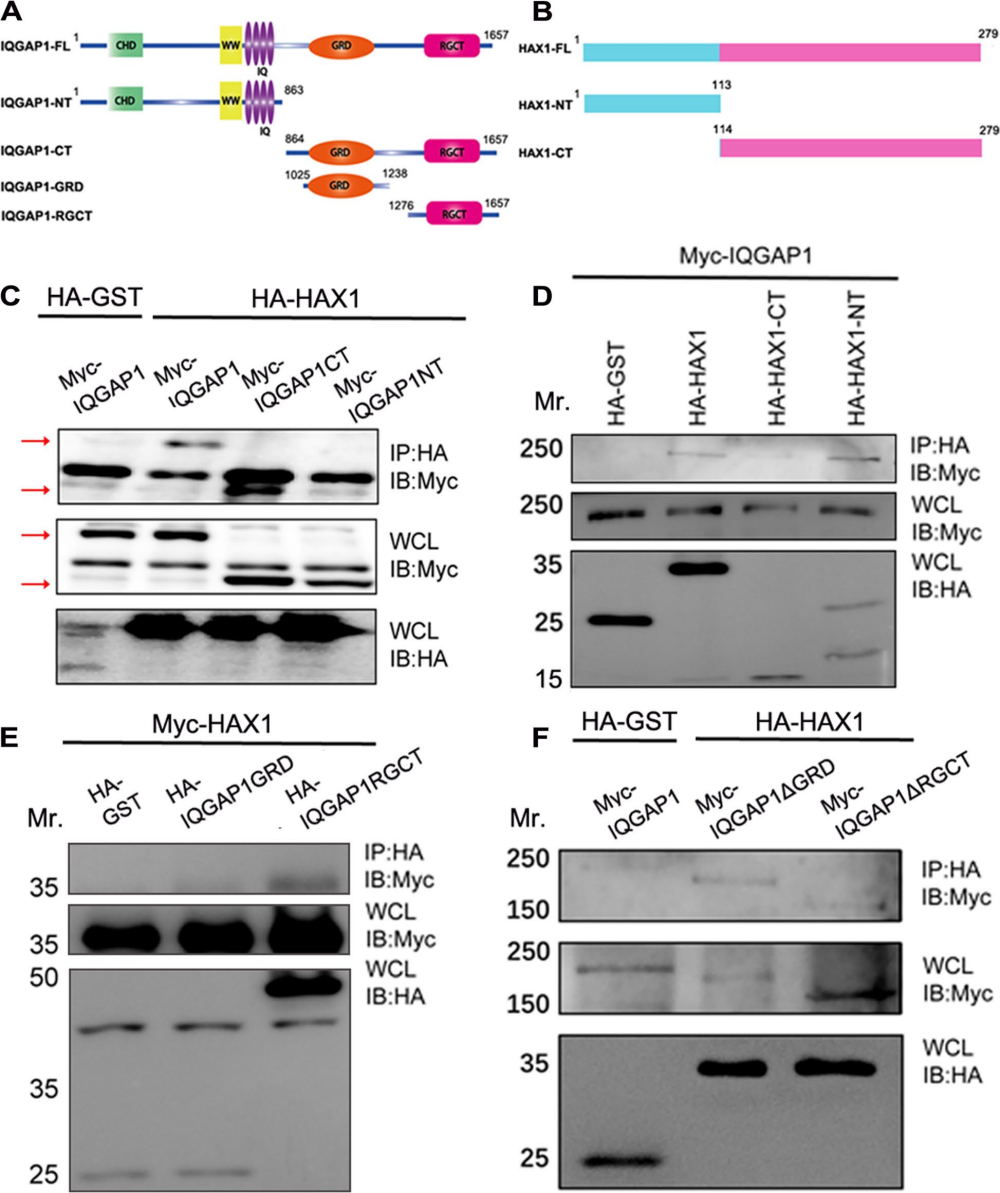
Biochemical characterization reveals a physical link between the Hax1 C terminus and IQGAP1-CT. **A**, Schematic of IQGAP1 and various IQGAP1 mutants used for coimmunoprecipitation assays. Residue numbers at domain boundaries are indicated. **B**, Schematic of Hax1 and various Hax1 mutants used for coimmunoprecipitation assays. Residue numbers at domain boundaries are indicated. **C**, Hax1 binds to the IQGAP1 C terminus (CT) rather than N terminus (NT). HEK293T cells were transfected with different plasmids as indicated. Cell lysates were immunoprecipitated (*IP*) with HA-agarose beads and immunoblotted (*IB*) with different antibodies as indicated. For whole-cell lysate (*WCL*), 10 μg of total protein was used. Note that Hax1 specifically pulls down IQGAP1 CT. **D**, IQGAP1 binds to the Hax1 C terminus (CT) rather than N terminus (NT). The association of different Hax1 mutants with IQGAP1 was determined by coimmunoprecipitation as described above. For whole-cell lysate, 10 μg of total protein was used. Note that IQGAP1 specifically pulls down Hax1 CT. **E** and F, Hax1 binds to the IQGAP1 RGCT rather than GRD domain. The association of different IQGAP1 mutants with Hax1 was determined by coimmunoprecipitation as described above. For whole-cell lysate, 10 μg of total protein was used. Note that Hax1 specifically pulls down IQGAP1 RGCT domain. GRD, Ras GTPase-activating protein-related domain; RGCT, RasGAP C-terminus domain

IQGAP1 is a scaffold protein with multiple domains [18]. To examine which domain contributes to the physical interaction with Hax1, we generated several deletion mutants according to its structure features (Fig. 2A). We first introduced deletion mutations to IQGAP1 by either retaining the 1–863 amino acids (IQGAP1-NT) or removing them (IQGAP1-CT) and carried out additional round of co-immunoprecipitation assays to determine which region is critical for its association with Hax1. As shown in Fig. 2C, C-terminal part other than the N-terminal part of IQGAP1 is essential for the interaction with Hax1. To narrow down the potential domain involved in the interaction between IQGAP1 and Hax1, we further generated and examined different IQGAP1 C-terminal truncation and deletion mutants, including the GRD and RGCT mutants and the ΔGRD and ΔRGCT mutants. Co-immunoprecipitation results show that RGCT, other than GRD is responsible for IQGAP1 association with Hax1 and deletion of RGCT abolished Hax1 interaction (Fig. 2E and F), suggesting that the RGCT is the critical domain for IQGAP1 interaction with Hax1. To further verify which region of Hax1 is essential for its interaction with IQGAP1, we also generated truncation mutants of Hax1 and carried out co-immunoprecipitation assays. As shown in Fig. 2D, C-terminal part other than the N-terminal part of Hax1 is essential for the interaction with IQGAP1. Together, our results suggest that C-terminal region of Hax1 and RGCT domain of IQGAP1 are the most critical binding determinants.

### Hax1 is essential for recruiting IQGAP1 to MTs

**Fig. 3:**
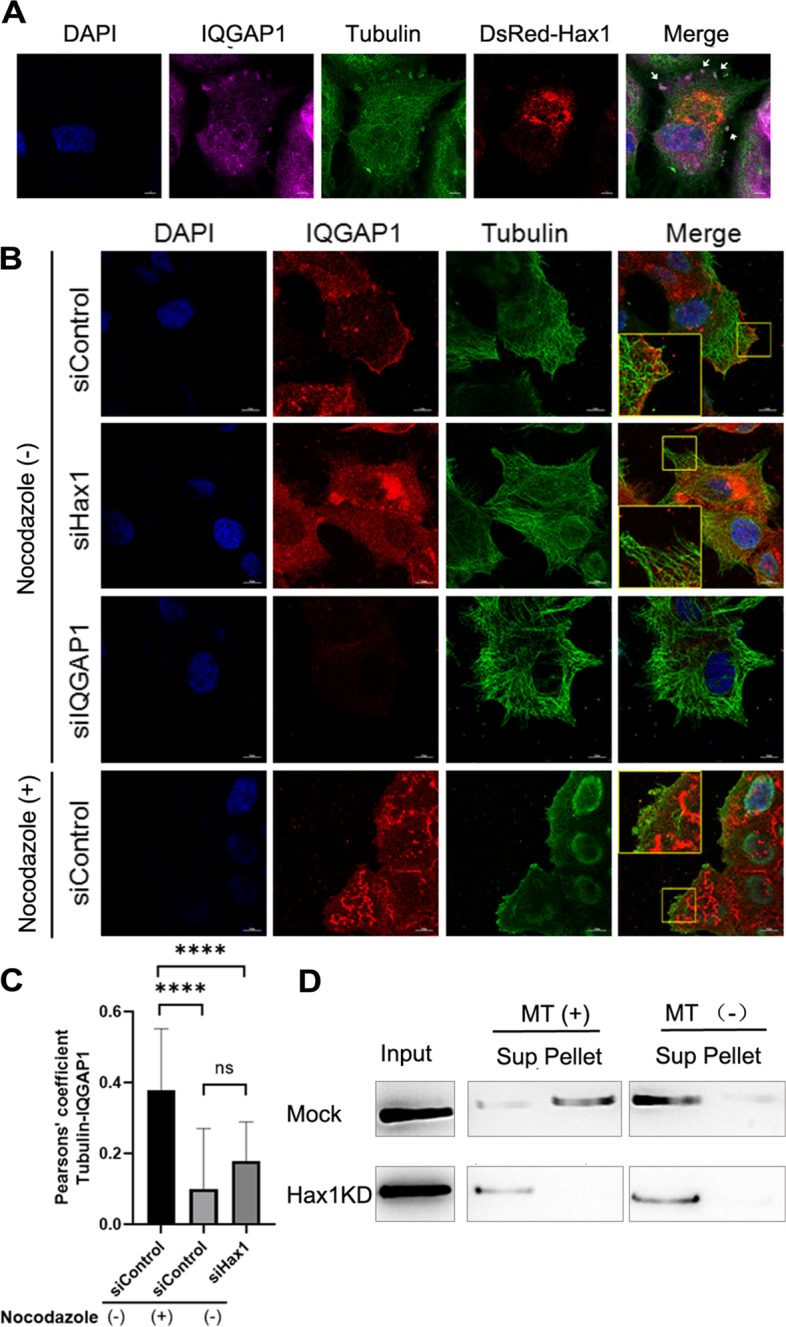
Hax1 recruits IQGAP1 to microtubules and the leading edge of migrating cells. **A**, MCF7 cells were transfected with DsRed-Hax1 and labeled with antibodies against tubulin (green), and IQGAP1 (magenta). Scale bar, 10 μm. Note that white arrows indicate the membrane ruffle-like localization of Hax1 is superimposed onto that of the IQGAP1 signal and the three channels are merged. **B**, Immunofluorescence staining for tubulin (green) and IQGAP1 (red) in MCF7 cells transfected with the indicated siRNAs and followed with or without treatment of nocodazole (10 μM) for 30 min. Cells were imaged by super-resolution N-SIM microscopy. The boxed areas are magnified as insets. Scale bar, 10 μm. **C**, Co-localization of IQGAP1 and MT (tubulin) was determined by Pearson correlation coefficient (*n* = 20 cells for each). Note that Hax1 localization with MT is reduced significantly in both nocodazole treated cells and Hax1-depleted cells (*p* < 0.0001). **D**, The interaction with microtubules (MT) was examined by co-sedimentation assay. The presence of proteins in the original lysate (Input), pellet of ultracentrifugation, as well as the supernatant (Sup) was determined by immunoblot with anti-IQGAP1 antibody

It is well documented that IQGAP1 accumulates at the polarized leading edge and areas of membrane ruffling [31, 32]. In our previous study, we have found that Hax1/EB2 interaction is critical for skin keratinocytes migration and EB2 can recruit Hax1 to microtubule growing ends [12]. The identification of IQGAP1 in the Hax1 protein complex prompted us to assume the role of Hax1 as an adaptor molecule to deliver key proteins such as IQGAP1 through microtubules to cell cortex. To examine the localization of Hax1-IQGAP1 protein complex around MTs, we performed immunofluorescence staining of MCF7 cells and imaged exogenously expressed Hax1 together with endogenous IQGAP1 and MTs. Super-resolution N-SIM imaging of stained MCF7 indicates a diffusely localized Hax1 throughout the cell, with enriched staining at the plasma membrane where IQGAP1 localizes. Moreover, this membrane ruffle-like localization of Hax1 is largely superimposed onto that of the IQGAP1 signal when signals from IQGAP1 are merged with Hax1 and MTs (Fig. 3A; white arrows). These data indicate that Hax1 and IQGAP1 colocalize predominantly at the MT plus ends near the leading edge of plasma membrane and may cooperate to promote cell migration.

To further test the potential relationship between IQGAP1 and MTs, we examined the effect of nocodazole on IQGAP1 localization. When cells were treated with nocodazole (10 μM) for 30 min, MTs were totally depolymerized. Under this condition, staining of IQGAP1 at the leading edge shows strongly diminished signals and co-localization between IQGAP1 and MTs is also significantly disrupted compared with the untreated control (Fig. 3B; quantification in Fig. 3C). Staining of Hax1-depleted cells shows a similar pattern with that treated with nocodazole: the localization of IQGAP1 at the leading edge becomes weaker and depletion of Hax1 disrupts the co-localization significantly (Fig. 3B; quantification in Fig. 3C). One possibility may explain this: Hax1 acts as a critical adaptor protein along MTs to increase the efficiency of IQGAP1 recruitment and/or contribute to maintenance of IQGAP1 at the leading edge. To further clarify the association between IQGAP1 and MTs, we performed MT pull down assays. The results indicate microtubule association of IQGAP1 in scramble siRNA-transfected cells but not in Hax1-depleted cells (Fig. 3D). Together, these results support the notion of a direct role of Hax1 coupled with MTs for recruiting IQGAP1 to the leading edge and membrane ruffling of cell.

### Hax1 is essential for IQGAP1 accumulation in FAs via MTs

**Fig. 4:**
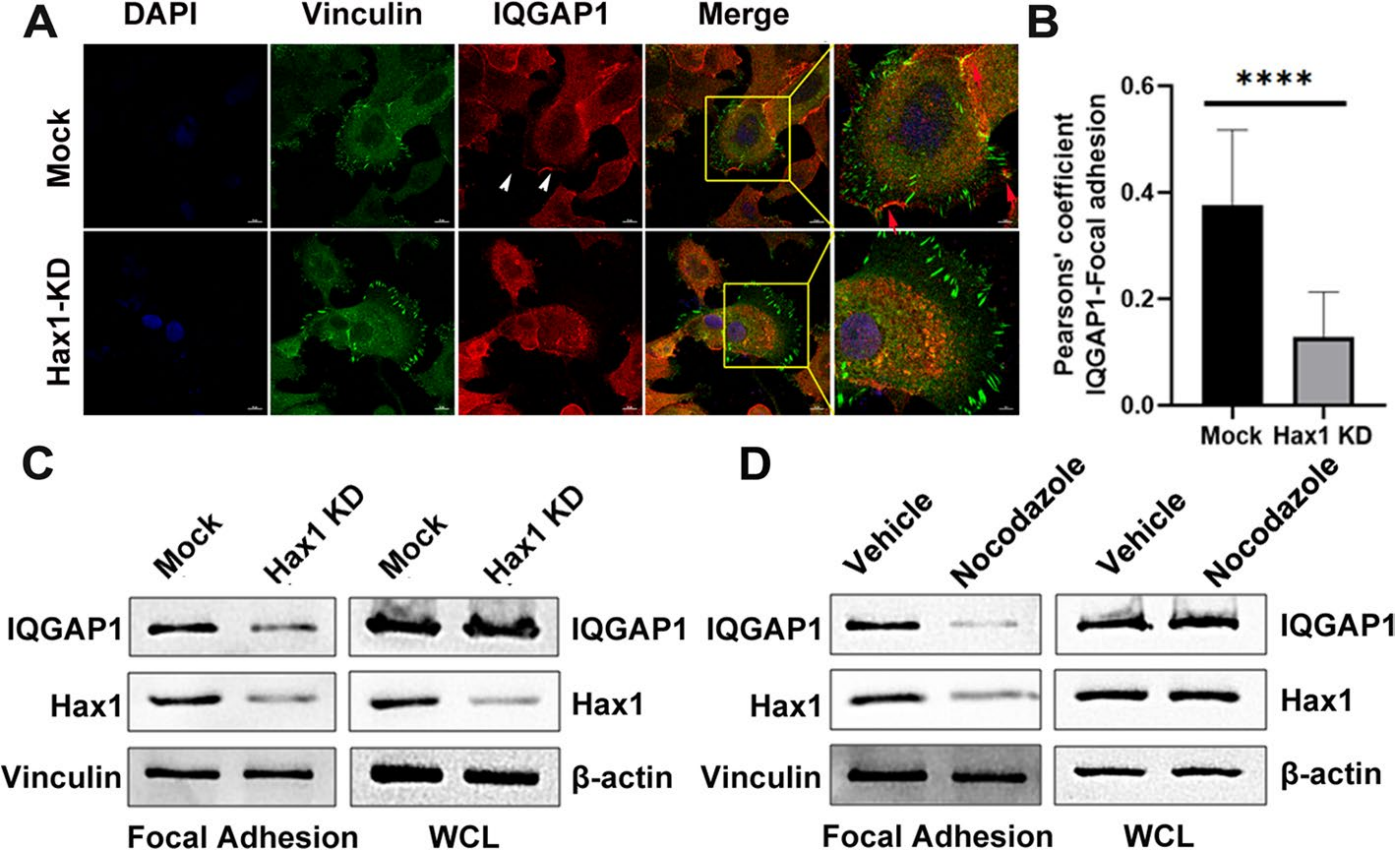
Suppression of Hax1 alters the subcellular location of IQGAP1 at FAs. **A**, Representative immunofluorescence images of mock or Hax1 knockdown MCF7 cells stained for Vinculin (green) and IQGAP1 (red). Cells were imaged by super-resolution N-SIM microscopy. The boxed areas are magnified as insets. White arrows indicate accumulation of IQGAP1 at the leading edge in control cells. Red arrows point at IQGAP1 localization at vinculin-positive FAs in control cells. Scale bar, 10 μm. **B**, Co-localization of IQGAP1 and FA (vinculin) was determined by Pearson correlation coefficient (*n* = 20 cells for each). Note that IQGAP1 co-localization with vinculin at the rim of the leading edge is reduced significantly in Hax1-depleted cells (*p* < 0.0001). **C**, Presence of IQGAP1 and Hax1 in isolated FAs or *WCL* (20 μg) from mock or Hax1 KD cells was determined by immunoblots. **D**, Protein abundance in isolated FAs or *WCL* (20 μg) from cells treated with or without nocodazole was determined by immunoblot with different antibodies as indicated

Previous findings demonstrated that peripheral FAs can be specifically targeted by MTs [7, 8]. Additionally, IQGAP1 was identified in FAs [27, 28]. Now we’ve confirmed that Hax1 is essential for recruiting IQGAP1 to the leading edge and membrane ruffling of cell where MTs localized. Next, we examined the colocalization of IQGAP1 with FAs in absence of Hax1. Immunofluorescence experiments of cells revealed that IQGAP1 displayed mainly cytoplasmic localization, enriched at the leading edge of mock cells (Fig. 4A; white arrows) with a small portion of IQGAP1 found in FAs (Fig. 4A; red arrows) as previously described [24, 33]. In contrast, IQGAP1 was significantly reduced at the rim of the leading edge of Hax1-KD cells and depletion of Hax1 dramatically disrupts the co-localization with FAs (Fig. 4A; quantification in Fig. 4B). To further investigate the distribution of IQGAP1 in detail, we performed immunoblot analyses and compared IQGAP1 level in FAs in absence of Hax1. FA proteins were fractionated from mock or Hax1-KD cells by an established protocol [27]. Knockdown of Hax1 leads to a significant decrease in IQGAP1 level in FAs (Fig. 4C). As we’ve confirmed that Hax1 coupled with MTs for recruiting IQGAP1 to the leading edge of cells (Fig. 3) and our previous study also identified the critical role of the association between Hax1 and a + TIPs–-EB2 in regulation of FA dynamics [12], suggesting the role of MTs in transporting IQGAP1 to FAs in a Hax1-dependent manner. Similarly, FA proteins were fractionated with or without prior treatment of nocodazole and subjected to immunoblots. Consistent with our hypothesis, significant diminished level of IQGAP1 in FAs was derived from nocodazole-treated cells (Fig. 4D). These results strongly suggest that Hax1 acts as an essential adaptor, permitting MT delivery of IQGAP1 to FAs, probably through interacting with EB2.

### The IQGAP1-Hax1 interaction is essential in cell migration

**Fig. 5:**
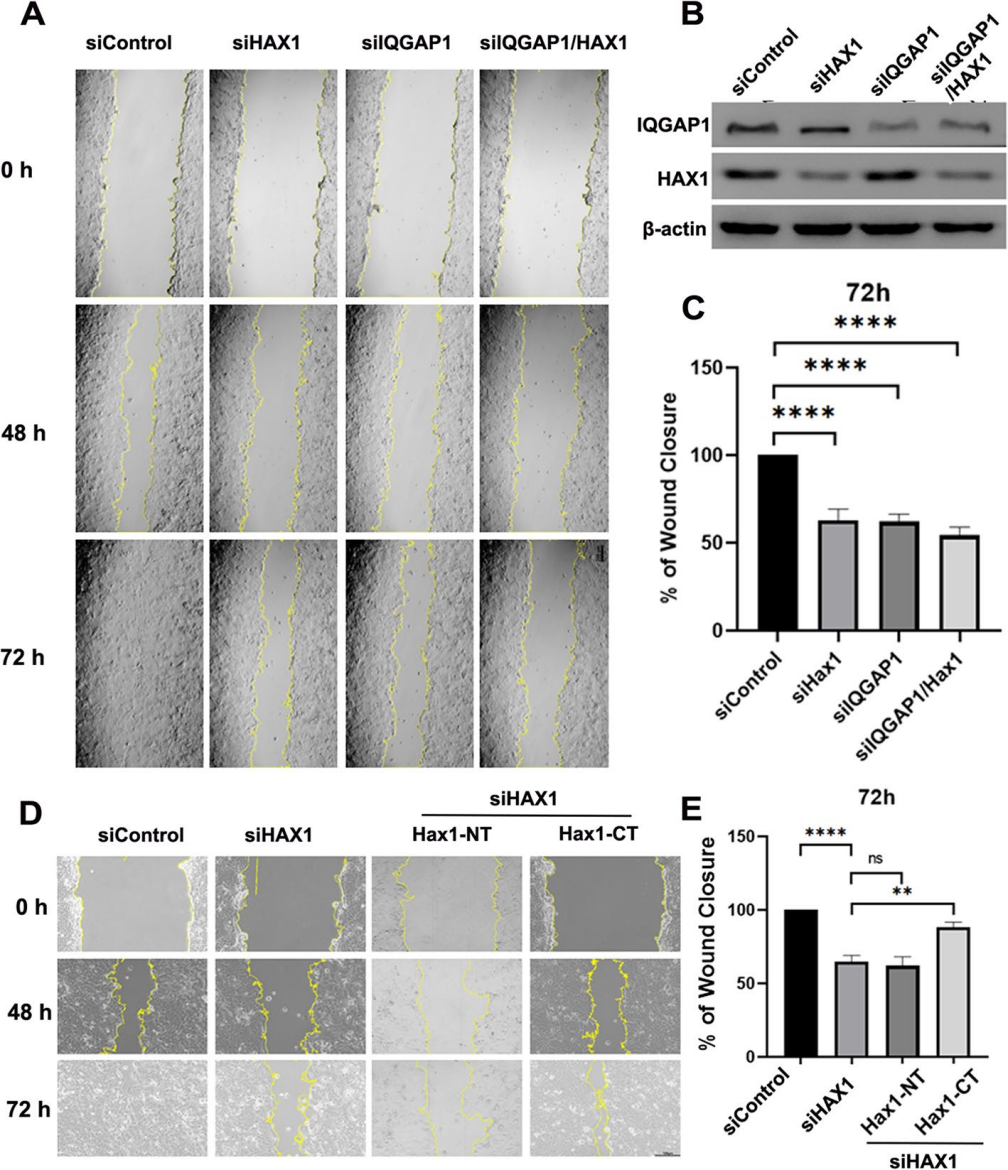
The IQGAP1-Hax1 interaction regulates cell migration. **A**, In MCF7 cells, suppression of Hax1, IQGAP1, or Hax1/IQGAP1 exhibits the same defect in directional migration. The migration of confluent monolayers of mock, Hax1 -depleted, IQGAP1-depleted, or Hax1/IQGAP1-depleted MCF7 cells were scratched, followed by visualization with phase-contrast microscopy at the indicated time points. **B**, Characterization of the efficiency and specificity of various siRNA oligonuclotides in MCF7 cells. The protein levels of Hax1, IQGAP1, or Hax1/IQGAP1 were significantly decreased in MCF7 cells transfected with corresponding siRNAs. **C**, The kinetics of in vitro wound healing in A are quantified. Note that suppression of Hax1, IQGAP1 or Hax1/IQGAP1 in MCF7 cells all lead to significant delay of in vitro wound healing (*n* = 3, *p* < 0.0001, Student’s *t* test). **D**, In MCF7 cells, directional migration of Hax1 knockdown cells and Hax1 –depleted cells rescued with different truncation mutants of Hax1 were evaluated by in vitro scratch wound assays. The migration of confluent monolayers of mock, Hax1 -depleted, or MCF7 cells co-transfected with Hax1 siRNA and two HA-Hax1 constructs (HA-Hax1-NT and HA-Hax1-CT) were scratched, followed by visualization with phase-contrast microscopy at the indicated time points. **E**, The kinetics of in vitro wound healing in D are quantified. Note that re-expression of Hax1-CT but not Hax1-NT mutant in Hax1-depleted MCF7 cells successfully rescues the defect of in vitro wound healing (*n* = 3, *p* < 0.01, Student’s *t* test)

Our previous work established an important role of Hax1 in regulation of cell migration in keratinocytes [12]. This study also confirmed that Hax1 is essential for cell migration in MCF7 cells and characterized a novel interaction between Hax1 and IQGAP1. To examine whether the involvement of Hax1 in cell migration is through the IQGAP1 process, we first monitored cell migration with Hax1 and IQGAP1 knockdown MCF7 cells. Western blotting analysis showed that Hax1 and IQGAP1 were efficiently depleted by the specific siRNA but not by scrambled sequences (Fig. 5B). In classical scratch wound healing assays, knockdown of Hax1 and IQGAP1 individually or simultaneously both displayed a significant delay in the recovery of these wounds (Fig. 5A). Hax1 knock down cells showed a similar decreased relative migration rate (~ 60% compared with that of the scrambled siRNA) with IQGAP1 cells, while double knockdown cells showed a even more decreased relative migration rate (Fig. 5C), indicating that Hax1 and IQGAP1 functions in cell migration by the same pathway to some extent.

As we’ve proved that C-terminal region of Hax1 is the most critical binding determinant for the IQGAP1-Hax1 interaction (Fig. 2), we next performed a rescue assay in which constructs expressing siRNA-resistant HA-tagged NT Hax1, and CT Hax1 were introduced into the confluent monolayers of MCF7 cells depleted of endogenous Hax1 by an siRNA targeted to Hax1. Classical scratch wound healing assays demonstrated that Hax1-depleted MCF7 cells displayed a significant delay in the recovery of wounds, and this migration defect can be rescued by Hax1-CT but not by Hax1-NT (Fig. 5D). Quantitative analyses showed that the migration speed was rescued to the comparable level of scramble siRNA-transfected cells only in the cells expressing Hax1-CT, while cells expressing Hax1-NT has no effect on rescuing this defect and its migration speed remained as the comparable level of Hax1 depleted cells (Fig. 5E), indicating that integration of the IQGAP1-Hax1 interaction is essential for directional cell migration.

### The IQGAP1-Hax1 interaction is essential in focal adhesion dynamics

**Fig. 6:**
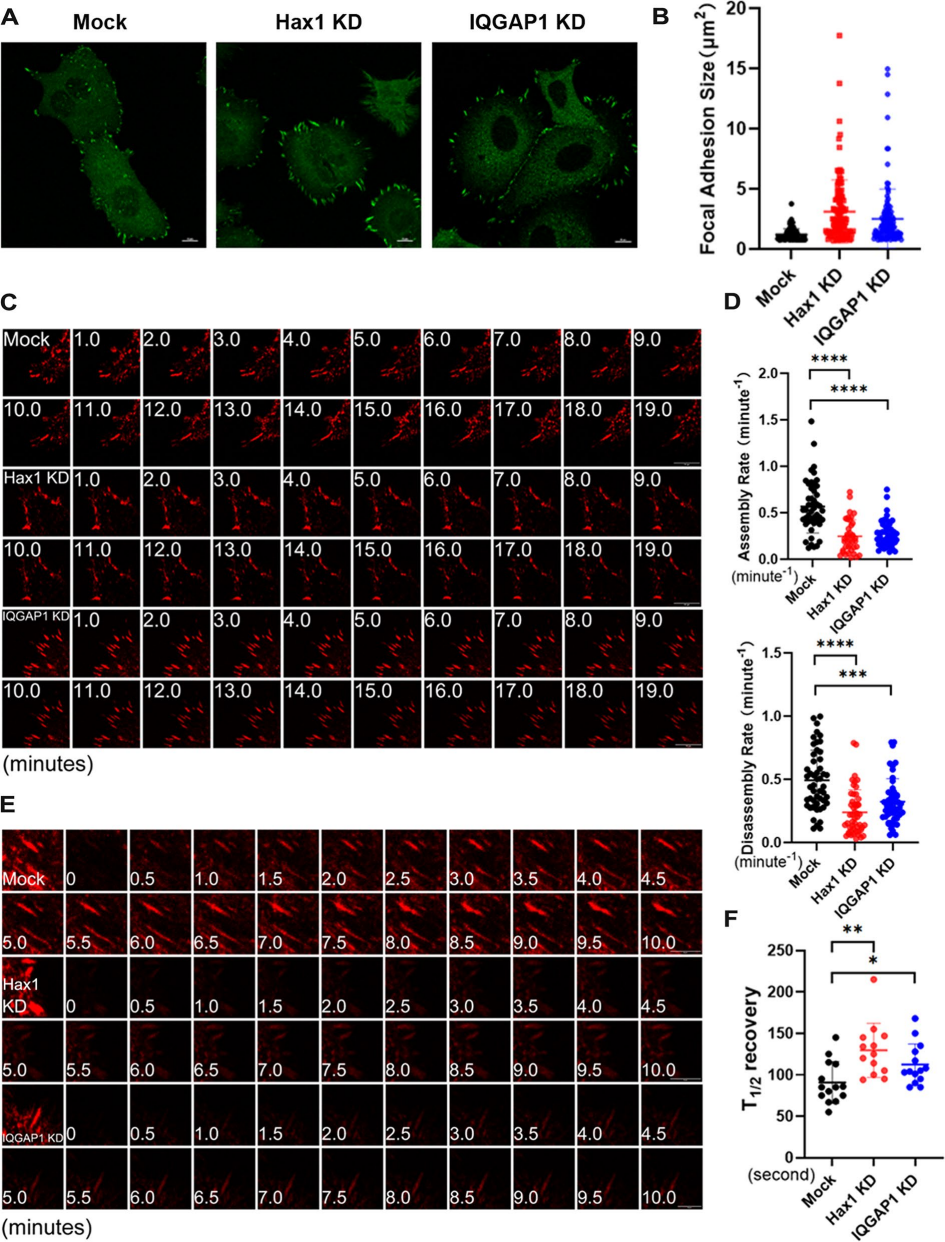
IQGAP1 interaction with Hax1 regulates focal adhesion dynamics. **A**, Representative immunofluorescence images of mock, Hax1 or IQGAP1 knockdown (KD) MCF7 cells stained for FA marker vinculin (green). Scale bar, 10 μm. **B**, Box and whisker plot indicating the size distribution of focal adhesions in mock, Hax1 or IQGAP1 KD cells (50 focal adhesions were analyzed for each genotype). Knockdown of Hax1 or IQGAP1 leads to a significant increase in focal adhesion size compared with mock cells (*p* < 0.0001 and *p* < 0.001 respectively, Student’s *t* test). **C**, Representative time-lapse images (montages) of DsRed-Zyxin-expressing MCF7 cells. Note the formation and dissolution of focal adhesions in mock cells and very static focal adhesion in Hax1 or IQGAP1 KD cells. Scale bar, 10 μm. **D**, Box and whisker plots revealing slow assembly and disassembly rates of focal adhesions in Hax1 or IQGAP1 KD cells relative to their mock counterparts. For each genotype, 50 focal adhesions were analyzed. Knockdown of Hax1 or IQGAP1 leads to a significant decrease of focal adhesion assembly and disassembly rate compared with mock cells (*p* < 0.0001 and *p* < 0.001 respectively, Student’s *t* test). **E**, FRAP was used to visualize reduced dynamics of FAs in mock, Hax1 or IQGAP1 KD cells Representative time-lapse images (montage) of FAs are shown. Scale bar, 10 μm. **F**, Box-and-whisker diagram quantifying the differences in half-time (T_1/2_) of FRAP between mock and KD cells (*n* > 10, *p* < 0.01 and *p* < 0.05 respectively, Student’s *t* test)

Our previous work established an important role of Hax1 in regulation of cell migration in keratinocytes through regulation of focal adhesion dynamics [12]. This study also confirmed that the IQGAP1-Hax1 interaction is essential for directional cell migration in MCF7 cells. To examine whether the effects of Hax1/IQGAP1 association on cell migration is through regulation of focal adhesion dynamics process, we first analyzed FAs size after *IQGAP1* or *Hax1* depletion. Immunofluorescence microscopy showed significantly enhanced labeling of focal adhesion in *IQGAP1* or *Hax1* knockdown cells relative to controls (Fig. 6A). Quantification of the presence of vinculin showed a significant increase in the size of focal adhesions in the two knockdown cells (Fig. 6B).

To directly address if FA dynamics were altered after *IQGAP1* or *Hax1* depletion, we performed confocal video microscopy to trace and examine the behavior of individual focal adhesion in knockdown cells by transfecting cells with plasmids encoding DsRed-Zyxin, a fluorescently labeled FA marker protein. Representative examples of the perturbations in focal adhesions dynamics arising from *IQGAP1* or *Hax1* depletion are shown in montages in Fig. 6C. During the interval of observation, FAs in control cells underwent continuous bouts of formation, maturation, and disassembly, whereas FAs in *Hax1* depleted cells were often static, which is in consistent with our previous reports in keratinocytes [12]. In line with our hypothesis, we have also observed significant more static FAs in MCF7 cells where IQGAP1 was down-regulated (Fig. 6C). Quantification of the kinetics of individual FAs revealed a dramatic decrease in both the assembly and disassembly rates of FAs in both knockdown cells (Fig. 6D). The defects in FA dynamics were further substantiated by fluorescence recovery after photobleaching (FRAP) experiments (Fig. 6E). *IQGAP1* or *Hax1* depletion resulted in a substantial increase in half-times of FRAP compared to controls (Fig. 6F). Exogenous expression of Hax1 mutant critical in IQGAP1 interaction (Hax1-CT) in the *Hax1* knockdown cells can restore focal adhesion turnover to some extent, whereas the other Hax1 mutant dispensable in IQGAP1 interaction (Hax1-NT) cannot (Fig. S2). Together, our results provide compelling evidence that IQGAP1 interaction with Hax1 plays a critical role in focal adhesion dynamics.

### Perturbation of IQGAP1-Hax1 interaction inhibits cell migration

**Fig. 7:**
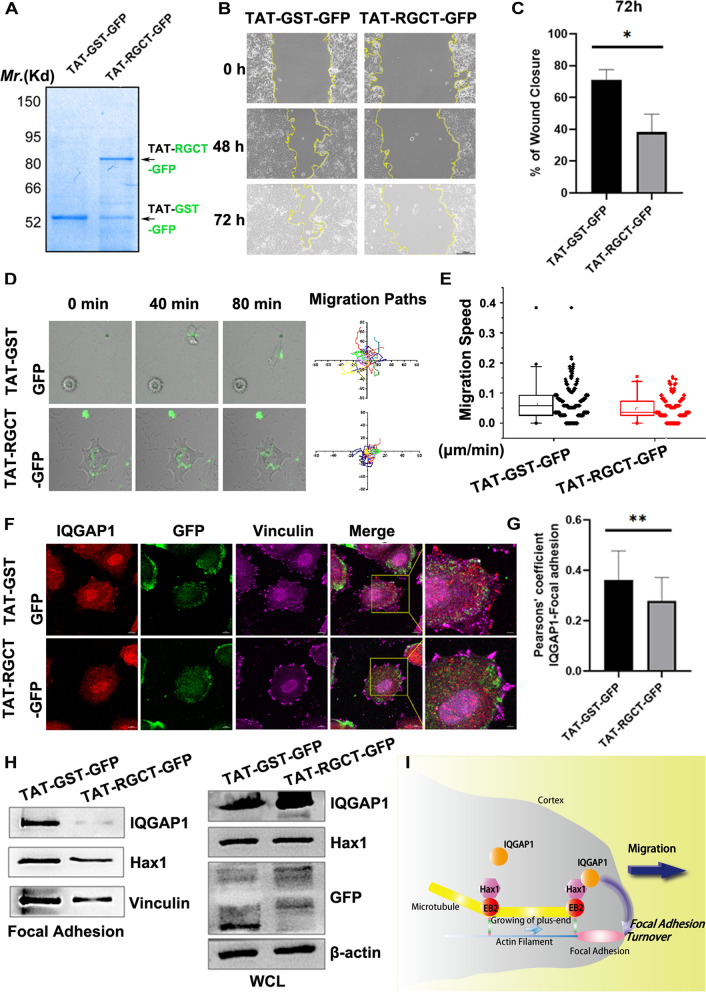
Perturbation of Hax1-IQGAP1 interaction inhibits cell migration. **A**, Coomassie Blue-stained SDS-PAGE gel was used to assess the quality of purified recombinant TAT-GST-GFP-His_6_ and TAT-RGCT-GFP-His_6_ proteins. *Escherichia coli* BL21(DE3) expressed TAT-RGCT-GFP peptide and TAT-GST-GFP (spike-in control) were purified with nickel-nitrilotriacetic acid affinity chromatography and desalted into DMEM. The respective TAT peptides are marked by *arrows.* **B**, Directional migration in MCF7 cells treated with TAT-GST-GFP or TAT-RGCT-GFP peptide were evaluated by in vitro scratch wound assays. Aliquots of TAT-GST-GFP or TAT-RGCT-GFP (2.5 μM) were added to confluent monolayers of MCF7 cells for 1 h and were scratched, followed by visualization with phase-contrast microscopy at the indicated time points. **C**, The kinetics of in vitro wound healing in B are quantified. Note that treatment of TAT-RGCT-GFP peptides leads to a significant delay of in vitro wound healing compared with the control peptides (*n* = 3, *p* < 0.05, Student’s *t* test). **D**, Real-time imaging of movements of individual MCF7 cells treated with TAT-GST-GFP or TAT-RGCT-GFP peptide. The migration paths of randomly picked transfected cells for each group are presented here as scatter plots (*n* = 20). **E**, Relative migration speeds in F are shown as box and whisker plots. Statistical analysis with Student’s *t* test showed that treatment of TAT-RGCT-GFP peptides leads to a significant decrease in speed compared with the control peptides (*p* < 0.01, Student’s *t* test). **F**, Representative immunofluorescence images of MCF7 cells treated with TAT-GST-GFP or TAT-RGCT-GFP peptide were stained for Vinculin (magenta) and IQGAP1 (red). Aliquots of TAT-GST-GFP or TAT-RGCT-GFP (2.5 μM) were added to cultured MCF7 cells for 1 h followed by fixation, immunocytochemistry and imaged by super-resolution N-SIM microscopy. Scale bar, 10 μm. **G**, Co-localization of IQGAP1 and FA (vinculin) was determined by Pearson correlation coefficient (*n* = 20 cells for each). Note that IQGAP1 co-localization with vinculin at the rim of the leading edge is significantly decreased in MCF7 cells treated with TAT-RGCT-GFP peptides compared with the control peptides (*p* < 0.01). **H**, Protein abundance in isolated FAs or *WCL* (20 μg) from MCF7 cells treated with TAT-GST-GFP or TAT-RGCT-GFP peptide (2.5 μM) was determined by immunoblot with different antibodies as indicated. **I**, Proposed working model accounting for the Hax1 function in directional cell migration. In short, the Hax1-IQGAP1 complex serves as a novel link to orchestrate directional cell migration via affecting focal adhesion dynamics

As we’ve proved that RGCT domain of IQGAP1 is the most critical binding determinant for the IQGAP1-Hax1 interaction (Fig. 2), we next construct a cell permeable peptide composed of the HIV-1 Tat protein transduction domain attached to the RGCT domain of IQGAP1 (TAT-RGCT-GFP) to disturb the endogenous IQGAP1-Hax1 interaction and further examine the functional importance of this interaction. The TAT-GST-GFP protein was constructed in the same way and used as the negative control. Both His-tag recombinant proteins were purified with nickel-affinity agarose beads and resolved in SDS-PAGE (Fig. 7A). Our pilot experiments showed that the internalization of TAT fusion proteins was seen in virtually all cells at 1 h after the addition of 2.5 μm TAT fusion protein, while 1 μm TAT fusion proteins lead to a much lower internalization rate (~ 50%). Thus, we next sought to test whether perturbation of IQGAP1-Hax1 interaction with TAT-RGCT-GFP protein affects directional migration. Classical scratch wound healing assays demonstrated that MCF7 cells treated with TAT-RGCT-GFP protein displayed a significant delay in the recovery of wounds compared with cells treated with TAT-GST-GFP control protein (Fig. 7B; quantification in Fig. 7C). To further determine whether the IQGAP1-Hax1 interaction is required for individual cell motility, we used video microscopy to monitor the velocities of individual cells treated with the TAT protein. Real-time imaging showed that cells treated with TAT-RGCT-GFP displayed quite limited moving trajectories compared with the TAT-GST control (Fig. 7D). Quantitative analyses show that TAT-RGCT-GFP significantly reduced the velocity of individual moving cell compared with the control (Fig. 7E), indicating that perturbation of IQGAP1-Hax1 interaction also impairs single cell motility.

To further determine whether the TAT-RGCT-GFP protein perturbs the co-localization between IQGAP1 and peripheral FAs, we carried out immunofluorescence staining of endogenous IQGAP1 together with vinculin in MCF7 cells pre-treated with TAT-RGCT-GFP or TAT-GST-GFP protein. As shown in Fig. 7F, IQGAP1 signal in cells treated with TAT-RGCT-GFP was more prone to distribute mainly in the endoplasm, while cells treated with the control TAT-GST-GFP displayed significant enrichment of IQGAP1 at the rim of the leading edge of cells. Quantitative analyses showed that co-localization between IQGAP1 and vinculin at peripheral FAs was significantly decreased by TAT-RGCT-GFP treatment but not by the control protein (Fig. 7G). To further investigate the distribution of IQGAP1 in detail, we performed immunoblot analyses and compared IQGAP1 level in FAs treated with TAT peptides. As expected, TAT-RGCT-GFP treatment leads to a significant decrease in IQGAP1 level in FAs compared with the control peptide (Fig. 7H). All of these phenocopied the loss of the Hax1-IQGAP1 complex, prompting us to examine if FA dynamics were altered after treatments. In line with our hypothesis, we have also observed significant FA elongation (Fig. S3A; quantification in Fig. S3B) and more static FAs (Fig. S3C; quantification in Fig. S3D) in MCF7 cells treated with TAT-RGCT-GFP peptides compared with the control peptides. Together, these results indicate that perturbation of IQGAP1-Hax1 interaction impairs efficient FA dynamics, which leads to impaired directional migration.

Therefore, we conclude that the IQGAP1-Hax1 interaction affects directional cell migration via locally regulating FA dynamics.

## Discussion

Hax1 is a ubiquitous and multifunctional protein involved in regulating cell migration in various systems. For example, Hax1 is essential for efficient neutrophil motility by regulating the detachment of uropod, integrin-mediated cell adhesion in neutrophils [17]. Besides, Hax1 overexpression has been observed in several tumors [13–15], including breast cancer [16]. However, reports on the effect of Hax1 on breast cancer cell migration are not entirely consistent and somewhat contradictory. Our previous study has also confirmed that Hax1 depletion in skin keratinocytes impairs cell migration and skin wound healing [12]. We also characterized a novel protein–protein interaction between Hax1 and a MT plus ends-binding protein–-EB2, and found this interaction is critical in focal adhesion turnover and epidermal migration [12]. However, both Hax1 and EB2 depletion has no effect on MT dynamics and it remains unclear that How does the Hax1–EB2 complexes regulate FAs? Clearly the identification of downstream effectors will help to explain how Hax1 functions during cell migration. In this study, we first confirmed that Hax1 is essential for cell migration in MCF7 cell line. To probe deeper into the underlying mechanism, we carried out affinity purification coupled with LC–MS/MS and demonstrated a novel interaction between Hax1 and IQGAP1 that is involved in cell migration. Our results indicate that depletion of endogenous Hax1 or IQGAP1 or disruption of their interaction in vitro can diminish cell migration. We further provide evidence that Hax1 may regulate cell migration through affecting IQGAP1 cellular localization and its co-localization with peripheral FAs in the leading edge of cells and perturbation of Hax1-IQGAP1 interaction impairs FA dynamics which leads to impaired cell migration.

IQGAP1 is a multi-domain scaffolding protein that mediate protein–protein interactions and essentially participated in a number of cellular events including cell–cell adhesion and cell migration [20, 21]. Besides, IQGAP1 is overexpressed in a variety of cancers [34, 35]. Potentially, inhibitors of IQGAP1 functions could prevent tumor invasion, proliferation, and migration. For example, a study demonstrated that transgenic GLK promotes tumor metastasis and cell migration through IQGAP1. GLK-induced cell migration and lung cancer metastasis were abolished by IQGAP1 depletion [23]. Thus, preliminary studies targeting IQGAP1 are encouraging to expand upon the distinct roles of IQGAPs in physiology and cancer. Our results show that RGCT domain which locates in the C terminus of IQGAP1 is critical for its interaction with Hax1. Previous studies show that RGCT domain interacts with many MT plus end tracking proteins (+ TIPs) such as CLIP-170 and Clasp2 to regulate MT dynamics and orchestrate cell migration [25, 36]. Growing microtubules serve as tracks to deliver key factors to the cell cortex to participate in a number of cellular events. Although many + TIPs have been identified as IQGAP1-interacting proteins, more IQGAP1-interacting + TIPs remain to be identified and characterized and how they are specifically involved in regulating cell migration remain to be elucidated. Our previous study has demonstrated a novel interaction between Hax1 and EB2, another + TIPs, proving the potential association between Hax1 and microtubules [12]. It is reasonable that IQGAP-Hax1-EB2 signaling axis is also involved in the regulation of cell migration through dynamic membrane-microtubule interactions. In this study, our data suggest, as expected, Hax1 depletion significantly diminished the IQGAP1 signals at the leading edge of cell, mimicking the effect observed after MT disruption by nocodazole, suggesting that Hax1 is essential for recruiting IQGAP1 to the cell cortex via MTs, probably through interaction with EB2. Our previous research revealed that the C terminus other than N terminus of Hax1 is necessary for the interaction with EB2 [12]. While in this study, we found that C terminus other than N terminus of Hax1 is also essential for the interaction with IQGAP1. These results demonstrate that C terminus of Hax1 is a critical part for association with both EB2 and IQGAP1, serving as a linkage between MT plus-ends and the cell cortex. Collectively, our studies identify Hax1 as novel IQGAP1-interacting + TIPs and add more evidence to the fact that RGCT domain of IQGAP1 is + TIPs binding region.

Apart from + TIPs, IQGAP1 also interacts with many membrane-resident proteins such as β-catenin, E-cadherin, and APC [24, 37, 38]. Moreover, it is worth to mention that IQGAP1 distributes at leading edges of migrating cells and was identified as the component of FA in recent studies [27, 28]. Additionally, IQGAP1 was found to interact with integrin linked kinase (ILK) that links integrins in focal complexes directly to the MT capture complex to stabilize the MT network [29]. However, it still remains elusive whether IQGAP1 is directly involved in regulation of FA dynamics. Growing evidence suggests the importance of endosomes for the local regulation of FA turnover and cell migration [39, 40]. For example, Schiefermeier et al., demonstated that MT plus end–directed transport of the p14–MP1 complex regulates localization and association of IQGAP1 to mature FAs and thereby controls FA dynamics [33]. They observed IQGAP1 accumulation in FAs upon loss of p14, which may be the cause of impaired FA dynamics because down-regulation of IQGAP1 by RNAi could rescue FAs and the migration defect initially observed in *p14*^−*/−*^ MEFs. However, in contrast with this previously published observation, we found that IQGAP1 knockdown not only caused significant delay in cell migration, which is in consistent with previous findings [26, 41], but also led to impaired FA dynamics. Perturbation of IQGAP1-Hax1 interaction significantly altered the subcellular localization of IQGAP1 from the rim of the leading edge to the endoplasm, decreased IQGAP1 level in FAs and impaired FA dynamics, mimicking the effect observed after loss of IQGAP1-Hax1 complex.

FAs turnover is precisely controlled by diverse signaling pathways. For example, MAPK signaling plays an important role in FA assembly as well as disassembly [42]. Rho family small GTPases are known to play a pivotal role in regulating FA dynamics and cell migration [43]. FAK and Src tyrosine kinases are also the two most prominent signaling molecules to be critically involved in cell motility and focal adhesion dynamics [44, 45]. While IQGAP1 was previously found to regulate the actin cytoskeleton, microtubules, and cell migration by multiple pathways including small GTPases and MAPK signaling through directly binding to the critical components of these signaling pathways [32, 46, 47]. So the phospho-regulation of FA protein(s) in the absence of the IQGAP1–Hax1 complex will in the future require large and comprehensive phosphoproteomics analyses. Also how IQGAP1-Hax1-EB2 axis as a whole functions and impacts these signaling pathways to regulate FA dynamics and cell migration should be evaluated in further studies.

In summary, based on our data we propose the following model. In migrating cells, IQGAP1 is localized to the cell cortex and partially localized to FAs. Hax1 acts as an important adaptor protein that directly interacts with IQGAP1 and + TIPs protein EB2 in its C terminus. The interaction between Hax1 and IQGAP1 is critical for recruiting IQGAP1 to the MTs and delivering IQGAP1 to FAs in the leading edge of cells, thereby affects FA dynamics and cell migration (Fig. 7I). Our findings not only provide critical insights into the role of Hax1 on migration of the epithelial cell layer in breast cancer cell lines, but also unravel a novel interaction between IQGAP1 and Hax1 and demonstrate its relevance in FA dynamics and cell migration. Moreover, our results demonstrate for the first time that Hax1 may serve as a critical adaptor to orchestrate MTs and FA turnover through directly interacting with + TIPs and FA component, thereby regulates cell migration. Finally, we give cues to the unresolved question that whether and how IQGAP1 is directly involved in regulation of FA dynamics. Further investigations are still required to test signaling pathways involved with IQGAP1-Hax1-EB2 axis and decipher the more sophisticated molecular mechanisms.

## Supplementary Information

**Additional file 1: Figure S1.**Hax1’s effect ondirectional cell migration.** Figure S2.** Hax1-IQGAP1interaction is essential for FA dynamics. **Figure S3.** Perturbation ofHax1-IQGAP1 interaction inhibits FA dynamics.

## Data Availability

The datasets used and/or analysed during the current study are available from the corresponding author on reasonable request.

## Abbreviations

ECM: Extracellular matrix
FA: Focal adhesion
MT: Microtubule
IQGAP1: IQ motif-containing GTPase-activating protein 1
GRD: Ras GTPase-activating protein-related domain;
RGCT: RasGAP C-terminus
EB: End-binding protein
