# Supplementary material for: Hax1 regulate focal adhesion dynamics through IQGAP1
Source: Cell Commun Signal. 2023 Jul 24;21:182. doi: 10.1186/s12964-023-01189-y (PMC10364419; doi:10.1186/s12964-023-01189-y)
Supplement: Supplementary file 2 — Additional file 1: Figure S1.Hax1’s effect ondirectional cell migration. Figure S2. Hax1-IQGAP1interaction is essential for FA dynamics. Figure S3. Perturbation ofHax1-IQGAP1 interaction inhibits FA dynamics. [file 12964_2023_1189_MOESM1_ESM.pdf]

Supplementary Figure Legends

Figure S1. Hax1's effect on directional cell migration

A, Suppression of Hax1 on the migration of confluent monolayers of MCF7 cells were assessed by *in vitro* scratch wound assays. Cells were treated with the indicated siRNAs for 48 h and then were wounded, followed by visualization using phase-contrast microscopy at the indicated time points.

B, Efficiency and specificity of Hax1 siRNA. The protein levels of Hax1 were significantly decreased in MCF7 cells transfected with corresponding siRNAs.

C, The kinetics of *in vitro* wound healing are quantified. Note that suppression of Hax1 in MCF7 cells leads to significant delay of *in vitro* wound healing (n = 3, $p <$ 0.01, Student's t test).

D, Movements of individual MCF7 cells transfected with the indicated siRNAs for 48 h were traced by video microscopyat 10-min intervals for 3 h . The migration tracks of randomly picked cells are shown here as scatter plots (n = 20).

E, Relative migration speeds in D are shown as box and whisker plots. Statistical analysis with Student's t test showed that suppression of Hax1 leads to a significant decrease in speed compared with control ($p < 0.01$).

Figure S2. Hax1-IQGAP1 interaction is essential for FA dynamics

A, Representative time-lapse images (montages) of DsRed-Zyxin-expressing Hax1 knockdown (KD) MCF7 cells exogenously expressing two Hax1 truncation mutants (Hax1-NT and Hax1-CT). Scale bar, 10 μm.

B, Box and whisker plots revealing assembly and disassembly rates of focal adhesions in Hax1 KD cells exogenously expressing two Hax1 truncation mutants (Hax1-NT and Hax1-CT). For each genotype, 50 focal adhesions were analyzed. Note that Hax1 CT but not the Hax1 NT mutant can rescue the defect in focal adhesion dynamics when introduced to express exogenously in the Hax1 KD cells. ($p < 0.01$ and $p < 0.001$ respectively, Student's t test).

Figure S3. Perturbation of Hax1-IQGAP1 interaction inhibits FA dynamics

A, Representative immunofluorescence images of MCF7 cells treated with TAT-GST-GFP or TAT-RGCT-GFP peptide (2.5 μM) stained for FA marker vinculin (magenta). Scale bar, 10 μm.

B, Box and whisker plot indicating the size distribution of focal adhesions in MCF7 cells treated with TAT-GST-GFP or TAT-RGCT-GFP peptide (2.5 μM) (50 focal adhesions were analyzed for each genotype). Cells treated with TAT-RGCT-GFP peptides leads to a significant increase in focal adhesion size compared with the control peptides ($p < 0.0001$, Student's t test).

C, Representative time-lapse images (montages) of DsRed-Zyxin-expressing MCF7 cells. Note the very static focal adhesion in cells treated with TAT-RGCT-GFP peptides. Scale bar, 10 μm.

D, Box and whisker plots revealing assembly and disassembly rates of focal adhesions in cells treated with TAT-RGCT-GFP or TAT-GST-GFP peptides. For each genotype, 50 focal adhesions were analyzed. Cells treated with TAT-RGCT-GFP peptides leads to a significant decrease of focal adhesion assembly and disassembly rate compared with the control peptides ($p < 0.05$ respectively, Student's t test).

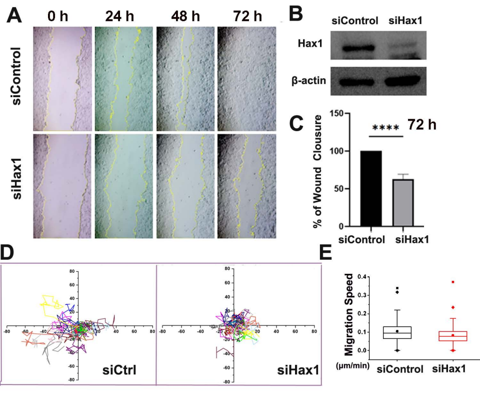

A
0 h
24 h
48 h
72 h
siControl
siHax1
B
siControl
siHax1
Hax1
β-actin
C
% of Wound Clousure
150
100
50
0
****
72 h
siControl
siHax1
D
siCtrl
siHax1
E
Migration Speed
(μm/min)
0.4
0.3
0.2
0.1
0.0
siControl
siHax1

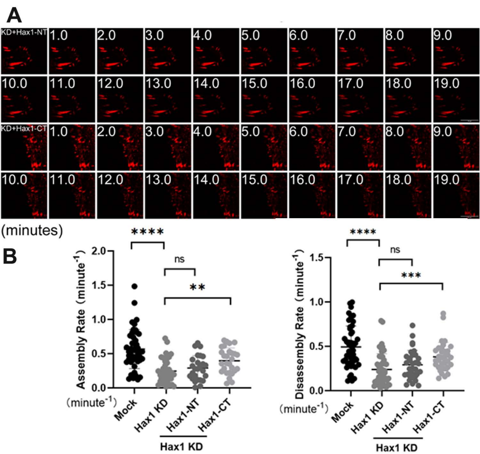

A
KD+Hax1-NT
1.0
2.0
3.0
4.0
5.0
6.0
7.0
8.0
9.0
10.0
11.0
12.0
13.0
14.0
15.0
16.0
17.0
18.0
19.0
KD+Hax1-CT
(minutes)
B
Assembly Rate (minute-1)
(minute-1)
Mock
Hax1 KD
Hax1-NT
Hax1-CT
Hax1 KD
****
ns
**
Disassembly Rate (minute-1)
***

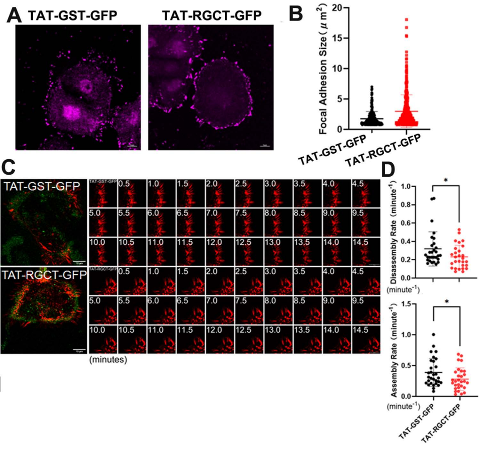

A
TAT-GST-GFP
TAT-RGCT-GFP
B
Focal Adhesion Size (μ m²)
20
15
10
5
0
TAT-GST-GFP
TAT-RGCT-GFP
C
TAT-GST-GFP
TAT-RGCT-GFP
0.5
1.0
1.5
2.0
2.5
3.0
3.5
4.0
4.5
5.0
5.5
6.0
6.5
7.0
7.5
8.0
8.5
9.0
9.5
10.0
10.5
11.0
11.5
12.0
12.5
13.0
13.5
14.0
14.5
(minutes)
D
Disassembly Rate (minute⁻¹)
(minute⁻¹)
Assembly Rate (minute⁻¹)
*
1.0
0.8
0.6
0.4
0.2
0.0
1.5
0.5
TAT-GST-GFP
TAT-RGCT-GFP
